# The protective properties of resveratrol against glycerol-induced acute kidney injury in rats

**DOI:** 10.22038/AJP.2024.24649

**Published:** 2025

**Authors:** Mohammadreza Baghishani, Soghra Mehri, Tahereh Aminifard, Amirhossein Jafarian, Hossein Hosseinzadeh

**Affiliations:** 1 *Department of Pharmacodynamics and Toxicology, School of Pharmacy, Mashhad University of Medical Sciences, Mashhad, Iran*; 2 *Pharmaceutical Research Center, Pharmaceutical Technology Institute, Mashhad University of Medical Sciences, Mashhad, Iran*; 3 *Department of Pathology, Ghaem Hospital, Mashhad University of Medical Sciences, Mashhad, Iran*

**Keywords:** Rhabdomyolysis, Acute kidney injury, Resveratrol, Inflammation

## Abstract

**Objective:**

After rhabdomyolysis, muscle tissue releases substances such as myoglobin, creatine kinase, and electrolytes into the bloodstream, potentially leading to acute kidney injury (AKI). Resveratrol (RSV) is a polyphenol compound with anti-inflammatory and antioxidant effects and it is found in various plants. This research evaluated the protective effects of RSV in rhabdomyolysis-induced AKI in rat kidneys.

**Materials and Methods:**

Thirty-six male Wistar rats were randomly assigned to six groups (n=6): 1) control (normal saline), 2) glycerol only (10 ml/kg, intramuscular), 3, 4, and 5) glycerol +RSV (5, 10, and 25 mg/kg, intraperitoneal injection) and 6) RSV (25 mg/kg). After 4 days, pathological alterations and the level of blood urea nitrogen (BUN) and serum creatinine were determined. Malondialdehyde (MDA), glutathione (GSH), neutrophil gelatinase-associated lipocalin (NGAL), and tumor necrosis factor-α (TNF-α) proteins were investigated in rat kidneys.

**Results:**

Injection of 50% glycerol (10 ml/kg, IM) resulted in pathological lesions, elevated levels of MDA (p<0.001), BUN (p<0.01), serum creatinine (p<0.001), TNF-α (p<0.01), and NGAL protein (p<0.001), and decreased GSH content (p<0.001) compared to the control animals. These findings indicated AKI induced by rhabdomyolysis. RSV (25 mg/kg) administration significantly decreased serum creatinine, BUN, MDA, NGAL, and TNF-α levels compared to the glycerol group. Histopathologically, tubule necrosis, myoglobin cast formation and glomerular atrophy increased in the glycerol group and reduced in animals that received RSV.

**Conclusion:**

In the glycerol-induced AKI rat model, RSV administration alleviated renal dysfunction by reducing oxidative stress and inflammatory responses.

## Introduction

Rhabdomyolysis is a serious condition where damaged muscle cells break down, releasing their contents into the circulation (Bosch et al., 2009; Melli et al., 2005; Zimmerman and Shen, 2013). Various factors can lead to rhabdomyolysis, including physical injuries like crush syndrome and earthquakes, as well as non-traumatic causes such as high body temperature, muscle oxygen deprivation, strenuous physical activity, and excessive alcohol consumption. It is important to identify the primary reason for rhabdomyolysis to provide appropriate treatment and prevent complications (Bosch et al., 2009; Melli et al., 2005; Zimmerman and Shen, 2013). 

About 10-40% of people who develop rhabdomyolysis will also experience acute kidney injury (AKI), but it is not clear if there is a specific treatment that targets the underlying factor of the condition (Bosch et al., 2009; Chander and Chopra, 2005; Chatzizisis et al., 2008). Currently, animal models of AKI induced by glycerol are frequently utilized by researchers to investigate the condition, as these models allow for controlled induction of the disease process without the confounding variables present in human studies (Mousleh et al., 2018). Injecting glycerol into the muscle triggers the release of myoglobin and other muscle components into the bloodstream, which finally leads to AKI. Research has revealed that the development of glycerol-induced AKI is a complex process involving multiple mechanisms, including myoglobin toxicity (Gburek et al., 2003; Reeder and Wilson, 2005), inflammation (Al Asmari et al., 2017), reactive oxygen species (ROS) production (Kim et al., 2010; Wu et al., 2017), and apoptosis (Homsi et al., 2010). 

A muscle protein called myoglobin which stores iron, is a key player in kidney problems caused by severe muscle breakdown (Holt and Moore, 2000; Panizo et al., 2015). The progression of rhabdomyolysis-induced AKI after the release of myoglobin from muscle cells is mediated by three distinct pathways: disruption of tubular function, damage to tubular epithelial cells due to oxidative stress, and constriction of renal blood vessels ( Holt and Moore, 2000; Panizo et al., 2015; Zager, 1989). During rhabdomyolysis, myoglobin is released into the bloodstream. It can be filtered out by glomeruli and reabsorbed into renal tubular cells through endocytosis, resulting in the characteristic brown color of urine (Bosch et al., 2009). Unbound myoglobin, not attached to any proteins or molecules, can directly harm renal epithelial cells and trigger inflammatory responses. When myoglobin accumulates in renal tubules, it can form insoluble precipitates with Tamm-Horsfall protein, leading to tubular occlusion (Zager, 1989). Iron content released from myoglobin following rhabdomyolysis can bind to oxygen and form a highly reactive compound called hydroxyl radical. This can damage mitochondria and induce programmed cell death (apoptosis) in renal tubular cells (Zager and Foerder, 1992). Myoglobin itself has an enzymatic activity that can speed up the breakdown of lipids, bringing about the formation of highly reactive compounds called isoprostanes. These can contribute to the overall oxidative stress experienced by the body during rhabdomyolysis (Reeder and Wilson, 2005). Iron accumulation and myoglobin-derived lipid peroxidation can damage the kidneys by triggering an excessive amount of cell death, known as ferroptosis, during rhabdomyolysis (Guerrero-Hue et al., 2019). It seems that oxidative stress plays a significant role in AKI caused by myoglobin. Therefore, the use of antioxidants in the treatment of this disease can be effective (Meghji et al., 2019; Luo et al., 2020). Resveratrol (trans-3,4',5-trihydroxystilbene; RSV) is a polyphenol belonging to the family of stilbenes. RSV is found in grapes, pistachios, and peanuts (Kitada and Koya, 2013; Malhotra et al., 2015). In various *in vivo* and *in vitro* studies, RSV has demonstrated various effects including antidiabetic (Crandall et al., 2012), neuroprotective (Turner et al., 2015), nephroprotective (Kim et al., 2011), antitumor (Oi et al., 2010), and cardioprotective (Kanamori et al., 2013) properties, as a consequence of its antioxidant, anti-inflammatory and cytoprotective activities (Malhotra et al., 2015). One of the important effects of RSV is its nephroprotective effects which have been observed in various nephropathies such as diabetic nephropathy (Palsamy and Subramanian, 2011), drug-induced renal injury (Do Amaral et al., 2008: Kim et al., 2011), and ischemia-reperfusion and sepsis-induced kidney injuries (Liu et al., 2015). 

Given the potent renoprotective effect of RSV observed in various models, the objective of this research was to evaluate whether RSV mitigates rhabdomyolysis-induced AKI in an animal model and to examine the potential mechanisms underlying this mitigation.

## Materials and Methods

### Chemicals

RSV was purchased from Naturalinbio, China. KCl, phosphoric acid and thiobarbituric acid (TBA) were obtained from Merck, Germany. From Sigma-Aldrich, Germany, 5, 5’-dithiobis-(2-nitrobenzoic acid) (DTNB) and trichloroacetic acid (TCA) were provided. Polyvinylidene difluoride (PVDF) was prepared from Bio-Rad, USA.

### Experimental design

Male Wistar rats, 210-230 g, were obtained from the animal breeding room of Mashhad School of Pharmacy and were housed in a coop under controlled conditions, including a temperature range of 22–25°C, a 12/12-hr light/dark cycle, and unrestricted access to water and food. To induce the AKI model, water access for the animals was withheld for 16 hr before glycerol injection. Each animal experiment was carried out following the guidelines that were set by the ethics committee of Mashhad School of Pharmacy (Ethical code: IR.MUMS.AEC.1401.054) and followed internationally accepted principles for animal use and care. Thirty-six male Wistar rats after a 2-day acclimation period, were set up into six groups (n=6). In Group 1 (control group), a volume of saline solution equivalent to 50% glycerol was administered intramuscularly in each hind leg. Group 2 (model group) received a single intramuscular injection of 50% glycerol (10 ml/kg), half of the glycerol dose was injected in each hind leg (Mousleh et al., 2018; Wu et al., 2017). Groups 3, 4, and 5 were treated with 50% glycerol (10 ml/kg), while half of the glycerol dose was injected in each hind muscle and different doses of RSV (5, 10, and 25 mg/kg) were administrated intraperitoneally for 4 days (Chander and Chopra, 2006; Osman et al., 2015). Group 6 received only RSV (25 mg/kg) via intraperitoneal injection (Osman, Telity et al. 2015) (Table 1). 

### Sample collection

At the end of the experimental period and in accordance with ethical standards, the rats were euthanized via decapitation 24 hr after the final treatment. Subsequent to euthanasia, serum and tissue samples were isolated for further analysis. The left kidney was immersed in a 10 % solution of neutral buffered formalin for histopathological examination, while the right kidney was stored at -80 °C for future analyses. 

### Biochemical analysis

To evaluate renal function, blood serum samples were examined in a lab to determine the amounts of BUN and creatinine.

### Histopathological examination

Formalin was used to fix kidney tissue samples for a minimum of 24 hr. Subsequently, they were passed through an autotechnicon system overnight. Tissue sections were sliced to a thickness of 2 μm using a rotary microtome, mounted onto glass slides, and colored with hematoxylin and eosin. Following staining, the slides were dehydrated, cleared, and cover-slipped. Finally, the prepared sample slides were examined under a light microscope to assess histopathological damage in the kidney tissues. The kidney tissues were examined for histopathological damages and assigned a score based on the following criteria: 1. Normal, 2. Mild damage (5-25% of tubules affected), 3. Moderate damage (26-50% of tubules affected), 4. Severe damage (50-75% of tubules affected), and 5. Extensive damage (over 75% of tubules affected) (Ramadhan et al., 2020).

### Measurement of lipid peroxidation

MDA is recognized as both a biomarker for lipid peroxidation and a significant indicator of oxidative stress. To evaluate MDA levels, the kidney tissues were homogenized at 4°C in a 1.15% KCl solution to prepare a 10% (w/v) homogenate. Initially, 3 ml of 1% phosphoric acid and 1 ml of 0.6% TBA solution were mixed with 0.5 ml of the homogenate. After boiling and cooling the mixture, 4 ml of n-butanol was added. Following centrifugation, the absorbance of the supernatant was measured at a wavelength of 532 nm using a spectrophotometer. The MDA content is presented in units of nmol/g tissue (Niehaus and Samuelsson, 1968).

### Determination of reduced glutathione (GSH) content

Moron et al. method was employed to quantify the level of glutathione (GSH) as an independent antioxidant compound in the kidneys (Moron et al., 1979). In this method, a reaction occurs between the free sulfhydryl groups and the 5,5'-dithiobis-(2-nitrobenzoic acid) (DTNB) reagent under alkaline conditions. Using phosphate buffer (pH 7.4) and trichloroacetate (TCA) solution (10%) in a 1:1 ratio, the tissues were homogenized (10%) and prepared. After centrifugation, 500 µl of the supernatant was mixed with 2.5 ml of phosphate buffer (pH 8) and 500 µl of DTNB reagent. The absorbance was recorded at 412 nm using a spectrophotometer. The GSH concentrations are presented in nmol/g tissue.

### Western blotting

This test was conducted following previously described methods (Vahdati Hassani et al., 2017; Vahdati Hassani et al., 2018). The protein from kidney tissue was extracted for TNF-α and neutrophil gelatinase-associated lipocalin (NGAL) analysis using western blotting. In summary, the kidney tissues were broken down in a buffer containing 50 mM Tris–HCl (pH 7.4), 2 mM ethylene glycol-bis(β-aminoethyl ether)- tetraacetic acid (EGTA), 1 mM Na3VO4, 10 mM NaF, 2 mM ethylenediaminetetraacetic acid (EDTA), 0.2% w/v sodium deoxycholate, 10 mM β-glycerol phosphate, 1 mM  phenylmethylsulfonyl fluoride (PMSF), 10% v/v 2-ME, and 2 μl protease inhibitor cocktail. Following the determination of the protein concentration in the supernatant using Bio-Rad Protein Assay Kit, equal volumes of the supernatants were then mixed with sodium dodecyl sulfate (SDS) sample buffer, heated, and loaded onto 12% SDS-PAGE gels. After running and transferring the proteins, the membranes were blocked with tris-buffered saline and tween 20 (TBST) containing 5% dry skim milk for 2 hr at room temperature. Afterward, the membranes were washed with TBST. Primary antibodies including TNF-α (Cell signaling, #3707), NGAL (Abcam#216462) and β-actin antibodies (Cell signaling #3700) were used according to the standard protocols. After washing with TBST, the blots were incubated with secondary antibodies (Cell Signaling, #7076, #7074) at a dilution of 1:3,000 for 2 hr at room temperature. Membranes were developed using enhanced chemiluminescence (ECL) Western Blotting Substrate and Alliance 4.7 gel doc (UK). The quantification of all band intensities was carried out using UVI-BandMap software (UVtec, UK) and normalized against the corresponding beta-actin intensities. 

### Statistical analysis

Analysis was performed statistically using GraphPad Prism 8.0 software (GraphPad Prism Software Inc.). The findings are presented as mean±SD. To compare the differences between the means, ANOVA followed by the Tukey-Kramer test was performed. To compare the BUN levels, the Brown-Forsythe and Welsh ANOVA and Dunnett’s T3 post hoc test were used to assess the statistical variations among the groups. Statistical significance was defined as p<0.05. Pathological markers are presented as median and interquartile range (IQR). Non-parametric statistical tests were employed to analyze the data. The Kruskal-Wallis test was used to compare multiple groups overall, and Dunn's multiple comparison test was used to identify specific group differences.

## Results

### Effect of RSV on kidney dysfunction induced by glycerol

**Figure 1:**
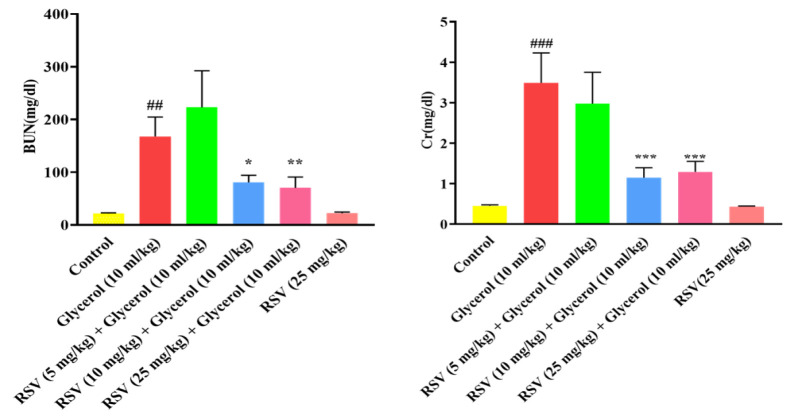
A: The effect of RSV on glycerol-induced changes in BUN. Data are expressed as mean±SD, (n=6). The Brown-Forsythe and Welsh ANOVA and Dunnett’s T3 post hoc test were used to assess the statistical variations among the groups. B: The effect of RSV on glycerol-induced changes in creatinine. Data are expressed as mean±SD, (n=6). Statistical difference was examined using the ANOVA test and a Tukey-Kramer posttest. ##p<0.01 and ###p<0.001 vs. the control group, and ***p<0.001, **p<0.01 and, and *p<0.05 vs. the glycerol group. RSV: Resveratrol

Administration of 50% glycerol (10 ml/kg, IM) significantly increased levels of serum creatinine (p<0.001) and BUN levels (p<0.01). Administration of RSV (10 and 25 mg/kg, for 4 days, IP) significantly decreased serum creatinine (p<0.001) and BUN levels (p<0.05 and p<0.01, respectively) compared to the glycerol group. Notably, administration of RSV alone (25 mg/kg) did not alter the mentioned factor levels when compared to the control group (Figures 1A and 1B).

### Effect of RSV on renal morphological alterations induced by glycerol

**Figure 2:**
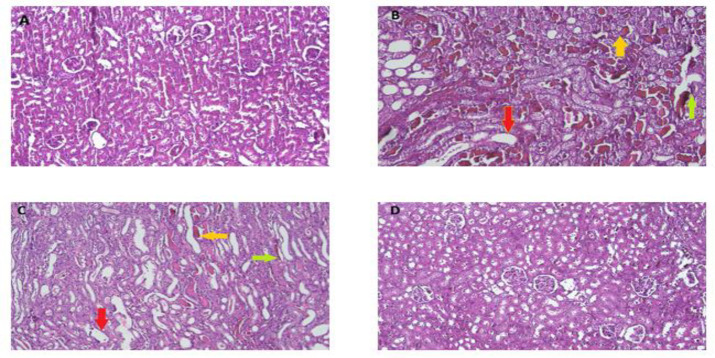
The effect of 50% glycerol (10 ml/kg) and RSV on histological changes in kidney morphology. The presented sections depict representative renal histopathology observed through hematoxylin and eosin staining at a magnification of 400x. A: control, B: glycerol, C: glycerol + RSV (25 mg/kg), and D: RSV (25 mg/kg). Red arrows indicate glomerular atrophy, yellow arrows indicate myoglobin casts and green arrows indicate tubular necrosis.

**Figure 3:**
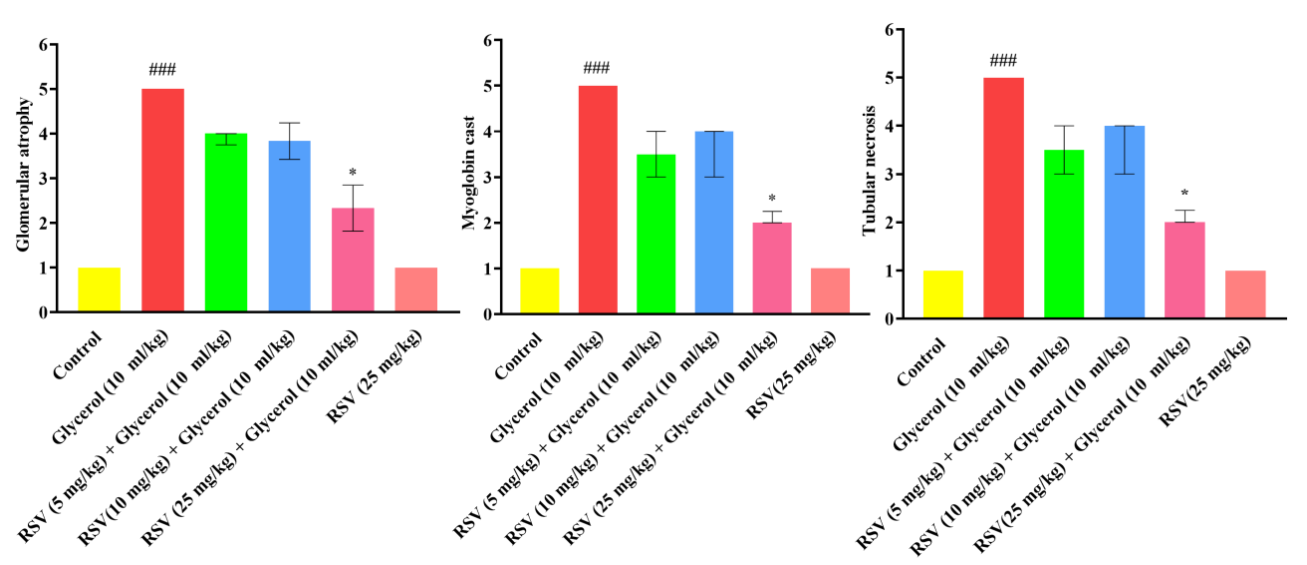
Effect of glycerol 50% (10 ml/kg) and RSV on different tissue indices. A: glomerular atrophy, B: myoglobin cast, and C: tubular necrosis. The data are shown as non-parametric (n=6). To assess the statistical difference, the ANOVA test and non-parametric Kruskal-Wallis test were employed. ###p<0.001 vs the control group, *p<0.05 vs. the glycerol group. RSV: Resveratrol

According to Figures 2 and 3, the injection of 50% glycerol (10 ml/kg) resulted in the development of pathological conditions, including tubule necrosis, myoglobin cast formation, and glomerular atrophy when compared to the control group (p<0.001). Conversely, concurrent administration of RSV with glycerol at a dosage of 25 mg/kg (IP) for 4 days led to a notable reduction in tubule necrosis, myoglobin cast formation, and glomerular atrophy, compared to the glycerol group (p<0.05). In animals that received RSV alone (25 mg/kg), normal kidney morphology was not altered when compared to the control rats.

### Effect of glycerol and RSV on lipid peroxidation induced by glycerol

**Figure 4:**
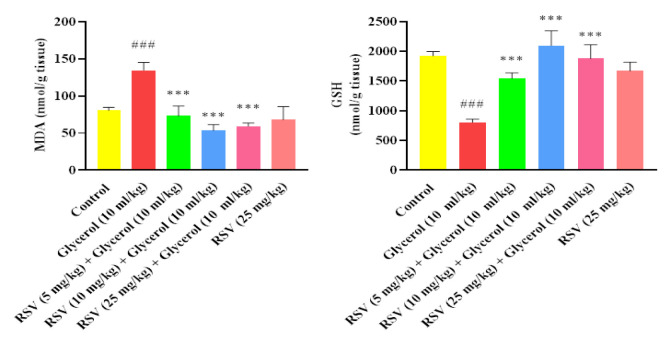
The impact of glycerol 50% (10 ml/kg) and RSV on the levels of A: MDA (malondialdehyde) and B: GSH (glutathione) in kidney tissue. Data are displayed as mean±SD (n=6). One-way ANOVA test and Tukey-Kramer post-test were used to investigate the statistical difference. ###p<0.001 vs. the control group, and ***p<0.001 vs. the glycerol group. RSV: Resveratrol

The results indicated that the administration of 50% glycerol (10 ml/kg, IM) is capable of increasing lipid peroxidation and elevating the amount of MDA in the kidney (p<0.001). In the kidney tissue, administration of RSV (5, 10, and 25 mg/kg, IP) along with glycerol after 4 days was effective in reducing the MDA content compared to the group receiving only glycerol (p<0.001). Interestingly, the administration of RSV (25 mg/kg) did not alter the MDA level compared to the control animals (Figure 4A).

### Effect of glycerol and RSV on glutathione (GSH) level in kidney

As illustrated in Figure 4B, the intramuscular administration of 50% glycerol (10 ml/kg) resulted in a significant decrease in the kidney’s GSH content (p<0.001). However, concurrent administration of RSV at doses of 5, 10, and 25 mg/kg (for 4 days, IP) led to a substantial increase in GSH content compared to the glycerol group.

### Effect of glycerol and RSV on the level of TNF-α and NGAL proteins in kidney tissues

**Figure 5:**
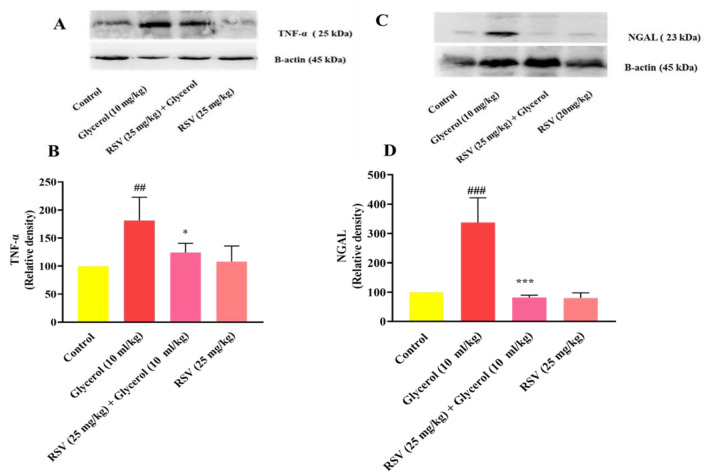
Effect of glycerol 50% (10 ml/kg) and RSV (25 mg/kg) on TNF-α and NGAL levels in kidney tissues. A and C: Represent immunoblot bands of the western blotting analysis; and B and D: Represent quantitative presentation of the immunoblots. Data is displayed as mean±SD (n=6). One-way ANOVA test and Tukey-Kramer post-test were used to investigate the statistical difference ##p<0.01 and ###p<0.001 vs the control group, and *p<0.05 and ***p<0.001 vs the glycerol group. RSV: Resveratrol, NGAL: neutrophil gelatinase-associated lipocalin

The levels of TNF-α and NGAL proteins in the kidney tissue increased significantly after the administration of 50% glycerol (10 ml/kg, IM), compared to the control rats (p<0.01 and p<0.001, respectively). The administration of RSV (25 mg/kg, for 4 days, IP) lowered the levels of TNF-α and NGAL proteins in the kidney tissue in comparison to the rats administered with glycerol alone (p<0.05 and p<0.001, respectively). Notably, after the administration of RSV (25 mg/kg) alone, the levels of TNF-α and NGAL proteins in kidney tissue remained at healthy levels compared to the control rats. (Figure 5).

## Discussion

In this study, we examined the protective effects of RSV on the kidneys against AKI induced by rhabdomyolysis in rats. Our study results demonstrated that the injection of 50% glycerol (10 ml/kg, I.M) led to changes in biochemical factors, increased oxidative stress markers, upregulated the expression of NGAL and TNF-α proteins, and consequently induced kidney damage. In contrast, RSV significantly reversed these mentioned factors. 

AKI induced by glycerol is a widely utilized animal model for evaluating nephroprotective agents, due to its well-defined pathophysiological mechanisms, including inflammation, oxidative stress, and programmed cell death (Chander and Chopra, 2005; Chander and Chopra, 2006; de Jesus Soares et al., 2007), which lead to rhabdomyolysis, myoglobinuria, and cast formation (Chander and Chopra 2005). 

Other studies have found that intramuscular injection of glycerol increased serum BUN and creatinine levels as signs of AKI (Wu et al., 2017). In the current study, 50% glycerol injection (10 ml/kg) increased BUN and creatinine levels, which is in harmony with the findings of other researchers. In research by Ramalingam et al. RSV (8 mg/kg, IP, for 28 days) in rats with nicotine-induced kidney damage significantly reduced creatinine and BUN levels in the serum, which indicates the improvement of kidney function (Ramalingam, et al., 2019). Also, RSV pretreatment (5 and 10 mg/kg, orally) reduced the level of creatinine and BUN in the kidneys of rats suffering from glycerol-induced acute renal failure (ARF) (Chander and Chopra 2006). In our research, alongside other studies, the renal protective effect of RSV was well evident. Its administration (10 and 25 mg/kg, IP, for 4 days) was able to reduce BUN and creatinine levels in serum. Consistent with our findings, various studies have revealed that the administration of glycerol causes histological changes in the kidney tissue. For instance, according to the research, the administration of 50% glycerol (8 ml/ kg, I.M) induces tissue damage, including interstitial inflammation, epithelial cell vacuolation and epithelial cell necrosis in the kidney tissue (Ramadhan et al., 2020). 

In our study, RSV (25 mg/kg, for 4 days, IP) reduced the pathological lesions such as tubular necrosis, myoglobin cast, and glomerular atrophy created following glycerol injection. The beneficial effects of RSV in healing pathological lesions have been proven in other studies, for example, RSV (25 mg/kg/day, orally, for 4 days) reduced kidney lesions including intraluminal casts, tubular cell necrosis, and tubular lumen dilation (de Jesus Soares et al., 2007). Likewise, RSV (5 and 10 mg/kg p.o) preceding the glycerol injection by 60 min, reduced histological changes such as tubular necrosis, hemorrhagic casts, hyaline casts, and apical blebbing caused by glycerol injection in the cortex and outer medulla of rats (Chander and Chopra 2006). 

Recent research suggests that myoglobin, a heme-rich protein found in muscles, is one of the main factors for the creation and progress of rhabdomyolysis-induced AKI (Giuliani et al., 2019). After muscle damage, myoglobin is rapidly filtered by glomeruli and absorbed by tubular cells (Gburek et al., 2003). Once inside these cells, myoglobin can change its chemical state, leading to the generation of harmful ROS and the promotion of lipid peroxidation (Moore et al., 1998; Zager and Foerder 1992, Zager and Burkhart, 1997). Free hemoglobin and/or iron can catalyze ROS formation through the fenton reaction, further exacerbating cellular damage (Boutaud and Roberts 2011). Moreover, myoglobin outside the cells in the tubular lumen creates granular casts that could potentially cause tubular blockage (Heyman et al., 1996). 

In the present study, 50% glycerol (10 ml/kg, I.M) increased the level of MDA and reduced the GSH content in the kidney tissue, consistent with the findings of other researchers (Liu et al., 2013). Nevertheless, the animals that received RSV (5, 10, and 25 mg/kg, for 4 days, IP) after the injection of glycerol exhibited a decrease in MDA and an increase in GSH content compared to the glycerol-treated animals. In past studies, it has been found that RSV (30 mg/kg, IP) in septic rats decreased the level of MDA and increased the content of GSH in the kidney tissue (Kolgazi et al., 2006). Also, RSV (30 mg/kg, 16 weeks, by gavage) reduced oxidative stress induced by high glucose in rats. This reduction was achieved through an increase in the activity of superoxide dismutase (SOD) and sirtuin 1 (SIRT1), as well as an elevation in catalase (CAT) protein levels. Furthermore, the content of MDA in the kidneys was decreased (Wang et al., 2017), which corresponds to the outcomes of the present study.

Rhabdomyolysis-induced AKI has been linked to inflammation caused by muscle cell injury. This damage releases immunostimulatory molecules that trigger the production of pro-inflammatory cytokines including TNF-α and interleukin-6 (IL-6), which in turn contribute to further kidney harm (Bosch et al., 2009; Nechemia-Arbely et al., 2008; Shulman et al., 1993; Nishida et al., 2015). In our study, 50% glycerol (10 mg/kg, I.M) increased the expression of TNF-α protein in the kidney tissue, which is in line with the results of other researchers (Yoon et al., 2008) have demonstrated that RSV (5 mg/kg/day, for 4 months, orally) in streptozotocin-induced diabetic rats significantly reduced inflammation by inhibiting pro-inflammatory cytokines like IL-1β, IL-10, TNF-α, and IL-6; also RSV improved mitochondrial function by reduction of renal 8-OHdG levels (Ma et al., 2016). Our findings proved that RSV (25 mg/kg, for 4 days, IP) decreased the level of TNF-α protein in the kidney tissue, which agrees with the results of other studies on the anti-inflammatory effects of RSV. 

NGAL is a protein found in various human tissues, such as the colon, trachea, lungs, and kidneys. It is a member of the lipocalin family of proteins. However, excessive levels of NGAL have been associated with certain diseases, such as AKI (Cowland and Borregaard, 1997). In addition, it has been found that the level of NGAL protein increases following the onset of AKI (Fauzi et al., 2016). In agreement with previous reports, the administration of 50% glycerol (10 ml/kg) raised the expression of NGAL protein in the kidney. In addition, Koca et al. have reported that in rats suffering from diabetes, the level of NGAL protein decreased after administration of RSV (25 mg/kg, for 4 weeks, IP) (Koca et al., 2016). In the present study, it was also found that the RSV (25 mg/kg, IP, for 4 days) markedly diminished the level of NGAL protein in the kidney tissue compared to the group receiving glycerol, which shows that RSV with its antioxidant and anti-inflammatory properties, can be a suitable treatment option to treat AKI. 

**Figure 6:**
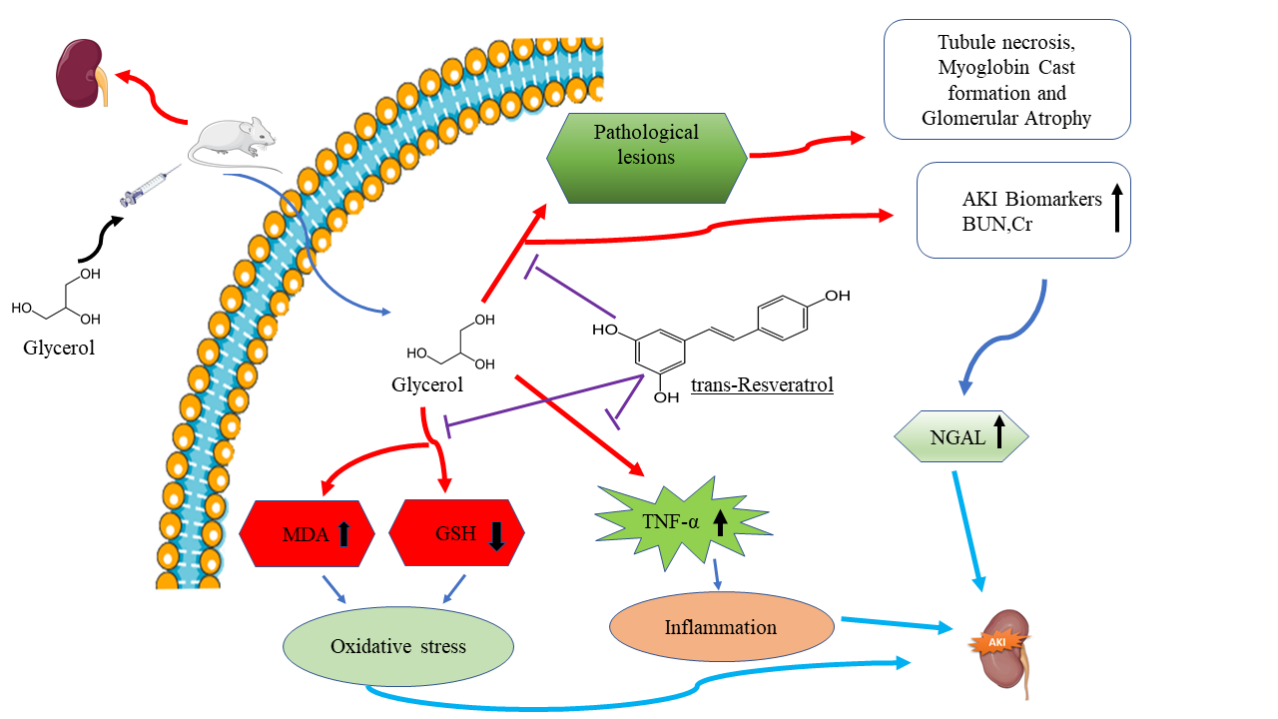
Simplified schematic of the mechanism of glycerol-induced AKI and the protective effect of the RSV in rats (Images from: smart.servier.com)

In conclusion, our research team’s findings demonstrate that 50% glycerol (10 ml/kg, I.M) induces rhabdomyolysis and subsequent AKI in rats. Our study reveals that RSV improves kidney function by normalizing levels of biochemical markers by reducing BUN and creatinine in the serum. Furthermore, RSV mitigates the pathological lesions resulting from glycerol injection through its antioxidant activity (lowering of MDA and elevating GSH levels) and its anti-inflammatory effects (reduction of TNF-α protein levels). Consequently, RSV appears to be an effective intervention against AKI caused by rhabdomyolysis (Figure 6).

**Table 1:** Study design: In groups 2 to 4, glycerol was injected intramuscularly as a single dose, and then groups 3, 4, and 5 received RSV intraperitoneally at doses of 5, 10, and 25 mg/kg for 4 days.

**Groups**	**Day 1**	**Day 2**	**Day 3**	**Day 4**	**Day 5**
Control	Saline (IM) + Saline (IP)	Saline (IP)	Saline (IP)	Saline (IP)	Sacrificed
Glycerol 10 ml/kg	Glycerol 10 ml/kg (IM)	-	-	-	Sacrificed
Glycerol 10 ml/kg + RSV 5 mg/kg	Glycerol 10 ml/kg (IM) + RSV 5 mg/kg (IP)	RSV 5 mg/kg (IP)	RSV 5 mg/kg (IP)	RSV 5 mg/kg (IP)	Sacrificed
Glycerol 10 ml/kg + RSV 10 mg/kg	Glycerol 10 ml/kg (IM) + RSV 10 mg/kg (IP)	RSV 10 mg/kg (IP)	RSV 10 mg/kg (IP)	RSV 10 mg/kg (IP)	Sacrificed
Glycerol 10 ml/kg + RSV 25 mg/kg	Glycerol 10 ml/kg (IM) + RSV 25 mg/kg (IP)	RSV 25 mg/kg (IP)	RSV 25 mg/kg (IP)	RSV 25 mg/kg (IP)	Sacrificed
RSV 25 mg/kg	RSV 25 mg/kg (IP)	RSV 25 mg/kg (IP)	RSV 25 mg/kg (IP)	RSV 25 mg/kg (IP)	Sacrificed

RSV: Resveratrol; IP: Intraperitoneal; IM: Intramuscular
